# Montreal Veterinary College

**Published:** 1882-04

**Authors:** 


### MONTREAL VETERINARY COLLEGE.

The annual commencement of the Montreal Veterinary College took place on March 30. Prof. Osler addressed the graduates, giving them some excellent advice as to their future, urging them to continue their studies and to take notes of everything as they went through life, that being the only way great discoveries in science were made. The following gentlemen having fulfilled the requirements of the curriculum and passed the successful examinations, written and oral, in botany, chemistry, physiology, cattle pathology, and practice of veterinary medicine and surgery, received the diploma of the eollege:

A. J. Chandler, Coaticook, Que.; Walter Wardle, Montreal; Alexander Glass, Philadelphia; Fred Torrence, Montreal; C. B. Robinson, Middlemarch, Ont.; Joseph M. Skally, Boston; D. E. P. Campbell, St. Hilaire, Que.; Paul Paquin, St. Andrews, Que.; Olivier de Maisonneuve, Terrebonne, Que.; Philias F. Labelle, St. Dorothee, Que.; Pierre Gadbois, Terrebonne, Que.

The following prizes were distributed: Silver medal for best general examination, A. J. Chandler; special prize for general proficiency, Alexander Glass; microscope for practice of veterinary medicine and surgery, A. J. Chandler. Second prize, Fred Torrence, B.A. Obstetrics and Cattle Pathology—First prize, A. J. Chandler; second, Walter Wardle. Anatomy—First prize, Walter Wardle; second, A. J. Chandler. Entozoa prize, won by Fred Torrence; Dentistry prize won by C. B. Robinson.

